# RGS14 Regulation of Post-Synaptic Signaling and Spine Plasticity in Brain

**DOI:** 10.3390/ijms22136823

**Published:** 2021-06-25

**Authors:** Nicholas H. Harbin, Sara N. Bramlett, Carolina Montanez-Miranda, Gizem Terzioglu, John R. Hepler

**Affiliations:** Department of Pharmacology and Chemical Biology, Emory University School of Medicine, Atlanta, GA 30322, USA; nicholas.hughes.harbin@emory.edu (N.H.H.); sara.bramlett@emory.edu (S.N.B.); carolina.montanez@emory.edu (C.M.-M.); gizeemterzioglu@gmail.com (G.T.)

**Keywords:** RGS protein, RGS14, synaptic plasticity, G protein, H-Ras, calcium, calmodulin, 14-3-3, nucleocytoplasmic shuttling, hippocampus

## Abstract

The regulator of G-protein signaling 14 (RGS14) is a multifunctional signaling protein that regulates post synaptic plasticity in neurons. RGS14 is expressed in the brain regions essential for learning, memory, emotion, and stimulus-induced behaviors, including the basal ganglia, limbic system, and cortex. Behaviorally, RGS14 regulates spatial and object memory, female-specific responses to cued fear conditioning, and environmental- and psychostimulant-induced locomotion. At the cellular level, RGS14 acts as a scaffolding protein that integrates G protein, Ras/ERK, and calcium/calmodulin signaling pathways essential for spine plasticity and cell signaling, allowing RGS14 to naturally suppress long-term potentiation (LTP) and structural plasticity in hippocampal area CA2 pyramidal cells. Recent proteomics findings indicate that RGS14 also engages the actomyosin system in the brain, perhaps to impact spine morphogenesis. Of note, RGS14 is also a nucleocytoplasmic shuttling protein, where its role in the nucleus remains uncertain. Balanced nuclear import/export and dendritic spine localization are likely essential for RGS14 neuronal functions as a regulator of synaptic plasticity. Supporting this idea, human genetic variants disrupting RGS14 localization also disrupt RGS14’s effects on plasticity. This review will focus on the known and unexplored roles of RGS14 in cell signaling, physiology, disease and behavior.

## 1. Introduction

### 1.1. RGS Protein Regulation of G-Protein Signaling

The family of the regulators of G-protein signaling (RGS) proteins regulate G-protein-coupled receptors (GPCRs) and G-protein signaling events [1,2]. GPCRs mediate cellular responses to a wide range of external stimuli, including peptide hormones, neurotransmitters, cytokines, sensory input, and many other natural ligands. Due to their regulation of nearly all physiological processes, GPCRs are ideal targets for many existing therapeutics and new drug discovery [3]. Agonist stimulation of GPCRs activates the associated heterotrimeric G protein (Gαβγ) by promoting guanine nucleotide exchange (replacement of GDP by GTP) on the Gα subunit, resulting in heterotrimer dissociation, thereby freeing Gα-GTP and Gβγ to activate their respective downstream effectors [4]. Gα subunits have intrinsic GTPase activity, which allows them to hydrolyze their bound GTP to GDP and terminate G-protein signaling. However, the intrinsic GTPase activity of Gα subunits is slow and cannot account for the precise temporal regulation of signaling events. RGS proteins bind directly to GPCRs and G proteins [1] to act as GTPase-activating proteins (GAPs) and markedly accelerate the hydrolysis of Gα-bound GTP to GDP, and the consequent deactivation of G-protein signaling [5,6].

### 1.2. RGS14 Interacting Partners

**Figure 1:**
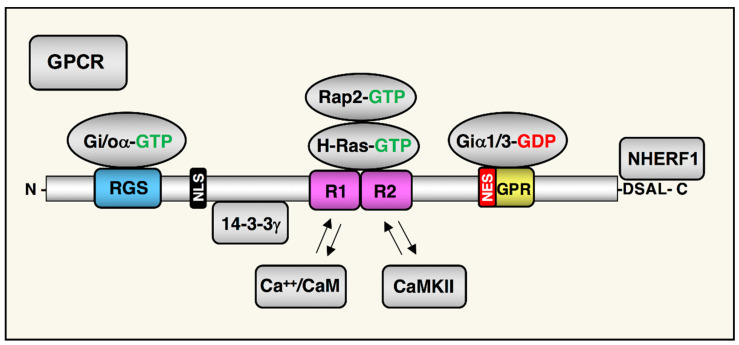
RGS14 structural organization and interacting partners. Regulator of G-protein signaling 14 (RGS14) is a ~62 kDa protein that contains three conserved domains and two motifs. The RGS domain serves as a GTPase activating protein (GAP) for active, GTP-bound Gαi/o proteins; the tandem Ras binding domains (R1/R2) enable interaction with H-Ras and Rap2; and the GoLoco/G protein regulatory motif (GPR) promotes binding to inactive, GDP-bound Gαi1/3 proteins and inhibits dissociation of GDP from Gαi1/3. RGS14 also contains a nuclear localization sequence (NLS) in the linker region between the RGS and R1/R2 domains and a nuclear export sequence (NES) embedded within the GPR motif. RGS14 interacts with 14-3-3 at phospho-Ser218 in the linker region between the RGS and R1/R2 domains, and also with calcium/calmodulin (Ca^2+^/CaM) and Ca^2+^/CaM kinase II (CaMKII) at undefined sites within the R1/R2 domain. Lastly, a PDZ-recognition motif (DSAL) is found at the C-terminus of human/primate (but not rodent) RGS14, which mediates RGS14 interactions with NHERF1.

While many RGS proteins are dedicated GAPs for target G proteins, some are more complex multifunctional proteins. One such complex RGS protein is RGS14, a ~62 kDa multi-domain scaffolding protein that is a member of the D/R12 subfamily of RGS proteins [7,8]. RGS14 serves as a GAP for Gi/o family members and can regulate their coupling to certain GPCRs [9,10,11,12], while both GPCRs and the non-receptor guanine exchange factor (GEF) resistance to cholinesterase inhibitor 8A (Ric8A) regulates RGS14–Gα–GTP complexes [12,13,14,15]. Besides acting as a GAP for certain Gα subunits, RGS14 also integrates G protein, mitogen activated protein kinase/extracellular regulated kinase (MAPK/ERK), and calcium signaling pathways in host cells. The RGS14 domain structure includes a conserved RGS domain that binds active Gαi/o–GTP [16], a tandem Ras/Rap (R1/R2)-binding domain (RBD) that binds activated H-Ras–GTP, Rap2–GTP and Raf kinases [9,16], and a GoLoco/G protein regulatory (GPR) domain that binds inactive Gαi1/3-GDP [17] (Figure 1). Additionally, RGS14 binds calcium/calmodulin (Ca^2+^/CaM) and Ca^2+^/CaM-dependent kinase II (CaMKII) within the R1/2 RBD [17] and binds the 14-3-3γ scaffolding protein in the linker region between the RGS and R1/2 RBD [18]. Unlike most scaffolding signaling proteins, RGS14 is also a nuclear–cytoplasmic shuttling protein that contains a nuclear export sequence (NES) embedded within the GPR motif and a nuclear localization sequence (NLS) located between the RGS and R1 domains [19,20,21]. The multitude of binding partners confer to RGS14 a capacity to regulate many key signaling pathways and cellular processes, and, in turn, for its actions and subcellular localization to be regulated. In this review, we will discuss the current state of the knowledge about RGS14 signaling function in the brain, in particular, as it relates to its role in post synaptic signaling, plasticity, and animal behavior.

## 2. RGS14 Protein Tissue Distribution

The RGS domain is evolutionarily conserved among vertebrate and invertebrate species [22]. RGS14 specifically is conserved in vertebrates such as chimpanzee, rhesus monkey, dog, cow, mouse, rat, chicken, zebrafish, and frog, and in invertebrates as an early homologue [23]. In rodents, where RGS14 has been primarily studied, it is expressed in the brain, heart, lungs, kidney, and spleen [7,24,25].

**Figure 2:**
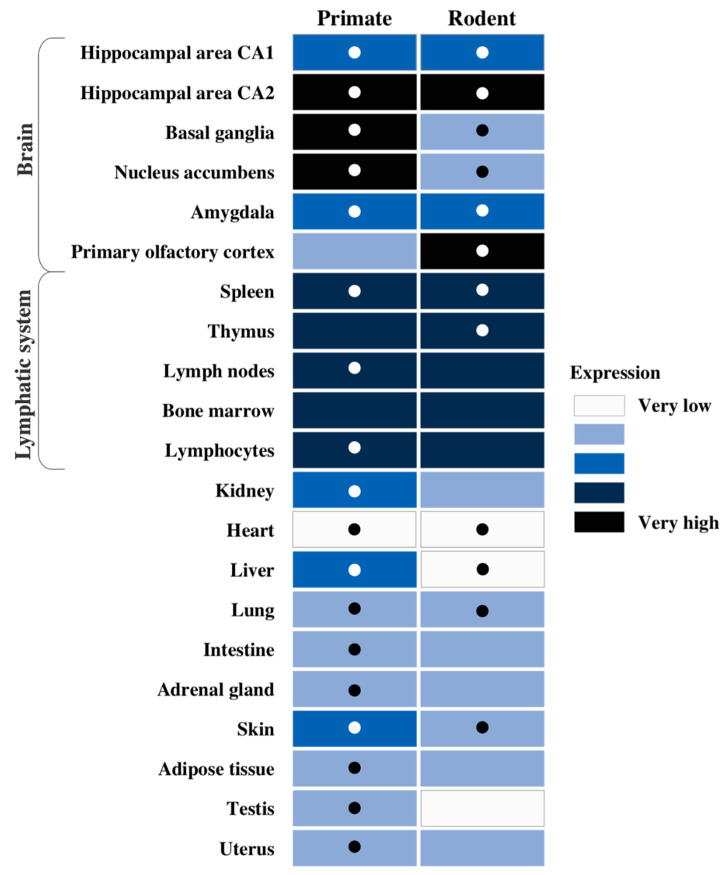
Reported tissue expression of RGS14 in adult primate and rodent. Cell shading indicates RGS14 mRNA expression across tissues. Dot indicates positive RGS14 protein expression data. Data were aggregated from the following sources: ProteomicsDB [49], Mouse Gene Expression Database (GXD) [50], Global Proteome Machine Database (GPMDB) [51], Allen Human Brain Atlas [52], Allen Mouse Brain Atlas [53], Human Protein Atlas [54], Ensembl [55], Evans et al. 2014 [27], Squires et al. 2018 [26], and Foster et al. 2021 [56]. Expression levels for mRNA were compiled from the sources cited above and defined in a relative manner from available data for each species (*M. musculus* and *R. norvegicus* for rodent, *H. sapiens* and *M. mulatta* for primate).

Within the rodent brain, the RGS14 protein is localized entirely to neurons and across cortical and limbic areas, with varying degrees of expression [26,27], although RGS14 mRNA is contained within the microglia and other brain-derived myeloid cell populations [28,29]. RGS14 mRNA and protein levels are developmentally regulated throughout postnatal development in mice, where RGS14 is upregulated until early adulthood, after which RGS14 levels drop and protein expression is more restricted [27]. In the adult brain, RGS14 is expressed in the hippocampus, piriform cortex, entorhinal cortex, layers II, III, and V of the neocortex, the anterior olfactory nucleus, and the orbital cortex [27]. More recently, we reported RGS14 expression in neurons of the central amygdala and the striatum [19]. In primates, including rhesus macaques and post-mortem tissue from humans [26], we find RGS14 protein expression in the caudate nucleus, putamen, and globus pallidus. We further elaborate on RGS14 expression within the brain in the next section (Figure 2).

RGS14 exists in peripheral tissues as well, where it plays an important role in physiology. RGS14 protein expression can be found in the ventricular tissue of healthy and hypertrophic mice [30]. In humans, RGS14 mRNA is found in the ventricular myocardium of patients with dilated and ischemic cardiomyopathy [31,32], while the RGS14 protein is detected in the left ventricle of healthy patients and those with dilated cardiomyopathy [30]. A recent study elucidated a novel role of RGS14 in the heart, where it was found to be downregulated in human failing hearts, murine hypertrophic hearts, and isolated hypertrophic cardiomyocytes [30]. Interestingly, its overexpression alleviated cardiac hypertrophy and dysfunction, suggesting RGS14’s involvement in cardiac remodeling [30].

Another emerging area of RGS14 peripheral action is the kidney. Recent genome-wide association studies (GWAS) have identified single nucleotide polymorphisms (SNPs) within the RGS14 gene locus associated with kidney dysfunction [33,34,35]. Subjects with genetic polymorphisms within and outside the RGS14 gene locus have higher susceptibility to nephrolithiasis [34,35,36,37,38], reduced glomerular filtration rates [33], and elevated levels of serum phosphate [39], all of which are indicative of kidney dysfunction [40]. Additionally, SNPs within the RGS14 gene are associated with elevated levels of parathyroid hormone (PTH) [41] and fibroblast growth factor 23 (FGF23) [42], which are both critical for proper kidney function [43]. Supporting this data, we have determined RGS14 expression in the kidney is high in human tissue, specifically in the proximal and distal tubule of the nephron [44], further suggesting a role for RGS14 in kidney function and disease.

Much less is known about RGS14’s role in the immune system. RGS14 RNA and protein are highly expressed in mouse lymphoid tissue and cells, including the thymus, spleen, lymph node, peritoneal cells, white blood cells, naïve B cells, and bone marrow [7,45,46,47], where it is downregulated in response to B-lymphocyte stimulation [45]. RGS14 is also expressed in THP-1 human monocytes/macrophages and in J774.A1 mouse macrophages, where it acts on aMB2 integrin during phagocytosis, through the RBD domain of RGS14 [48]. Recent transcriptome analysis of myeloid cells and microglia from the brain revealed RGS14 mRNA expression in subsets of brain-derived myeloid cells [28,29], indicating a role for RGS14 in immune function in the central nervous system as well as the periphery.

Recent studies reported RGS14 expression in mouse subcutaneous/inguinal white adipose tissue [22,25]. Surprisingly, loss of RGS14 expression in mice leads to a reduction in white adipose tissue and an increase in brown adipose tissue, resulting in improved metabolism and extended lifespan [22]. However, the mechanism of enhanced longevity in RGS14-KO mice and body regions, where RGS14 could be regulating metabolism and ageing, remains unknown.

While RGS14 clearly has roles outside of the brain, this review will focus on the physiological actions of RGS14 within the brain. The following discussions will summarize our current understanding of RGS14’s roles in postsynaptic signaling and plasticity at the cellular level, and RGS14’s roles in animal behavior.

## 3. RGS14 Expression in the Brain

### 3.1. RGS14 Developmental Expression

We have characterized the postnatal developmental expression of RGS14 in the mouse brain [27]. RGS14 mRNA levels are low and protein is absent in the brain at birth. RGS14 protein is first detectable in early postnatal development (P7), with whole-brain levels of protein and mRNA increasing across development (P7–P21) until reaching a plateau in adulthood that persists throughout maturity. In the primary olfactory areas, including the anterior olfactory nucleus and piriform cortex, and the olfaction-linked orbital and entorhinal cortices, expression is upregulated during development and maintained to varying degrees into adulthood. Transient postnatal expression is observed in neocortical layers II/III and V. Hippocampal protein levels are initially low at P7 before climbing markedly throughout development to show prominent expression in adulthood, specifically in area CA2 and the fasciola cinerea.

### 3.2. RGS14 Expression in Adult Rodent and Primate Brains

Adult rodents and primates share pronounced RGS14 expression in the hippocampus, striatum, and amygdala [26,27,56,57] (Figure 2). Notable levels of RGS14 are found in the primary olfactory areas in adult rodents, which are absent in primates [27]. Immunoblotting of hippocampal and striatal punches from mice and macaques revealed several apparent truncated RGS14 splice variants unique to the primate striatum [26].

RGS14 is strongly expressed in specific subregions of the hippocampus, notably in pyramidal cell bodies, axons, and dendrites throughout the entire rostro-caudal range of hippocampal area CA2 and in the fasciola cinerea. [26,27,57]. RGS14 staining is also observed in stratum oriens of area CA1, which are thought to be neuronal processes originating from CA2, and sparsely in CA1 pyramidal cells in the rodent brain. In the primate hippocampus, RGS14 exhibits typical expression in area CA2 [26], consistent with studies in mice [27,57]. However, RGS14 is also expressed in area CA1 of primates, where it is found pre- and post-synaptically in neuropil, pyramidal cell bodies, and proximal dendritic profiles [26]. Consistent with its expression in the hippocampus, RGS14 plays key roles in hippocampal-based learning and memory, and synaptic plasticity, which is to be discussed below.

Outside of the hippocampus, RGS14 is highly expressed in the basal ganglia, striatum, and amygdala in studies of primate tissue (macaque and human) [26]. The striatum as a whole is RGS14-rich, with the dorsal regions (caudate nucleus and putamen) exhibiting mildly higher expression levels than the ventral striatum (nucleus accumbens). Within the nucleus accumbens, RGS14 is present in both the core and shell subregions in rodent, and co-localizes with striatal-enriched protein tyrosine phosphatase (STEP) [56]. The morphology of RGS14-positive striatal cells is consistent with medium spiny neurons (MSNs), inhibitory projection neurons that innervate structures of the midbrain and basal ganglia. In primates, electron microscopy revealed that striatal RGS14 is largely located in the cytosol of postsynaptic structures, although significant staining is also found in the nucleus [26]. Immunoblot data suggest that striatal nuclear localization may be attributable to a truncated RGS14 splice variant with disrupted nuclear export functionality, though additional studies are needed for confirmation.

In addition to the striatum, several other basal ganglia structures exhibit RGS14 immunoreactivity, including both the internal and external segments of the globus pallidus and the substantia nigra pars reticulata [26]. RGS14 labeling in these regions is localized presynaptically in the axons and terminals of putative striatopallidal and striatonigral projection neurons. An additional population of presumably excitatory RGS14-containing inputs of yet-unknown origin is also found in the substantia nigra pars reticulata. Another region enriched with RGS14 is the amygdala, in which expression is found in putative projection neurons of the basomedial, basolateral, and centrolateral nuclei [19,56]. Projections from the central nucleus of the amygdala may comprise the unidentified RGS14-positive innervation in the substantia nigra, though further study is required.

## 4. RGS14 in the Hippocampus

RGS14 has been studied most extensively in the hippocampus, which is necessary for many types of memory, including spatial, episodic, and social [58,59]. The hippocampus is divided into the following four major subregions: the dentate gyrus (DG), and areas CA1, CA2, and CA3. These regions are morphologically and functionally distinct, have unique molecular expression profiles, and are innervated by each other as well as receiving and projecting connections from extrahippocampal regions [60].

Much of hippocampal research has focused on components in the classical tri-synaptic pathway (DG > CA3 > CA1), where excitatory granule cells in the dentate gyrus project onto the excitatory pyramidal cells of area CA3, which then project onto excitatory pyramidal cells of CA1 [61]. Importantly, the synaptic strength of the connections from dentate gyrus to CA3, and CA3 to CA1 can be modulated in a process known as synaptic plasticity. Synaptic plasticity in the trisynaptic pathway is thought to be the underlying basis of hippocampal-dependent memory [60,61] and has received much attention since its initial discovery. However, the importance of area CA2 in relation to hippocampal function, physiology, and behavior has only recently been investigated [62,63,64,65,66].

Area CA2, similarly to its neighboring areas CA1 and CA3, contains a high density of glutamatergic pyramidal cells as well as several different types of GABAergic inhibitory neurons [66]. CA2 also receives connections from area CA3 (similar to CA1), and projects forward onto CA1 and back onto CA3 [67]. Similarly to CA1, CA2 also sends and receives numerous connections from several different extrahippocampal regions [66]. Recent discoveries have unraveled the importance of area CA2 as being essential for social and maternal memory [68,69], temporal coding [70,71], and regulating hippocampal excitability and epileptogenic processes [72,73]. CA2’s involvement in disease is a promising avenue of research, especially regarding autism spectrum disorders, given CA2’s proclivity for housing social memory [68,74,75,76].

Unlike neighboring CA1 and CA3, CA2 is unique in that pyramidal cells in this region do not support synaptic plasticity [57,65,77]. Upon high-frequency stimulation of CA3 Schaffer collateral projections, subsequent long-term potentiation (LTP) that is expressed in area CA1 is suppressed in CA2. LTP can be expressed at CA3–CA2 synapses via a variety of pharmacological and genetic manipulations [66] (discussed below), suggesting multiple, compensatory mechanisms that restrict LTP in CA2. On a molecular level, numerous studies report a mechanistic basis for CA2 LTP inhibition. One is RGS14, which will be discussed at length below. Others include a high capacity for calcium buffering and extrusion [78,79], a high density of inhibitory input onto CA2 pyramidal cells [80,81], the expression of plasticity-restricting perineuronal nets around excitatory pyramidal cells [82,83], and a set of plasticity-resistant signaling proteins [57,84]. Interestingly, molecules that restrict CA2 LTP, including RGS14, are developmentally regulated [27,69,82], perhaps reflecting their participation in a critical period akin to those found elsewhere in the brain [85,86]. However, the physiological and behavioral roles of CA2 are an active area of research and remain only poorly understood.

RGS14 has been identified as a key actor in area CA2 to suppress synaptic plasticity [57,79]. In hippocampal slices derived from wild-type mice, high-frequency stimulation of CA3 inputs onto CA2 in hippocampal slices has little effect on post-synaptic LTP in CA2, while hippocampal slices obtained from RGS14 knockout (RGS14-KO) mice exhibit robust LTP in area CA2 in the same paradigm. Post-synaptic potentiation of dendritic spines typically produces an enlargement of the dendritic spines [87], which reflects the insertion of α-amino-3-hydroxy-5-methyl-4-isoxazolepropionic acid (AMPA) receptors into the post-synaptic density to maintain potentiation [88]. Our lab recently reported that RGS14 inhibits the enlargement of spines following two-photon glutamate uncaging in CA2 dendrites [79]. In the same report, we also observed RGS14 inhibition of glutamate-induced calcium influx into single spines. CA2 dendritic spines from RGS14-KO mice readily enlarge with a coincidental increase in calcium influx after two-photon glutamate uncaging. Of note, viral delivery of RGS14 to CA2 neurons of KO mice “rescued” suppression of LTP, and completely blocked LTP and spine plasticity in CA1 neurons, where RGS14 is sparsely expressed. Taken together, these data suggest that RGS14 engages common signaling pathways shared by both CA2 and CA1 neurons, important for regulating synaptic plasticity, and support a central role for RGS14 as a master signaling node that regulates postsynaptic plasticity in pyramidal cells of the CA2 hippocampus.

## 5. RGS14 Regulation of Postsynaptic Signaling

RGS14 controls synaptic signaling due to its capacity to bind proteins that are essential for postsynaptic functions and plasticity. As outlined above, RGS14 is a complex, multi-domain scaffolding protein that binds specific Gα proteins, H-Ras/Rap2 GTPases, 14-3-3, Ca^2+^/CaM, and CaMKII (Figure 1). Each of these proteins has been shown to play important roles in postsynaptic signaling and plasticity [89,90,91,92,93], and RGS14’s role in postsynaptic signaling is outlined below.

### 5.1. RGS14 Regulation of GPCR–Gi/o Activation

**Figure 3:**
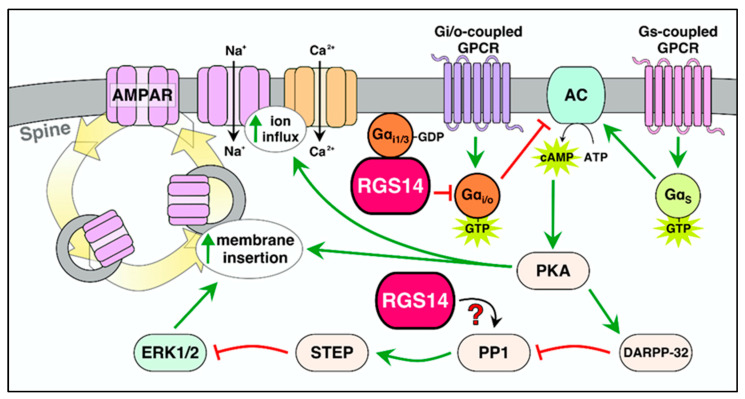
RGS14 modulation of G-protein-dependent signaling. RGS14 accelerates Gαi/o deactivation to limit GPCR–Gi/o signaling and disinhibit the adenylyl cyclase activity and downsteram cAMP signaling. Binding of inactive Gαi1/3–GDP localizes and anchors RGS14 to the plasma membrane, where it is optimally positioned to regulate the initial steps of intracellular post synaptic signal transduction. Likewise, it is possible that RGS14 modulation of GPCR signaling influences plastic processes such as AMPAR trafficking.

Tight spatial and temporal control of G-protein signaling is essential for proper synaptic and behavioral responses [92], and RGS14 is well positioned within the dendritic spines to regulate these processes (Figure 3). Many different neuromodulators activate Gi/o-coupled GPCRs in postsynaptic spines [3] to inhibit adenylyl cyclase activity, and decrease cAMP and PKA activity [94]. The RGS domain of RGS14 confers temporal negative regulation of Gi/o proteins activated by these receptors, thereby maintaining homeostatic control over cAMP and cAMP-dependent signaling (e.g., PKA activation) in the postsynaptic neuron [9,10,46]. RGS14–Gi/o interactions are dissociated by non-receptor GEF Ric8A, likely causing reactivation of Gi/o [13,14], although the importance of Ric8a or its interaction with RGS14 in postsynaptic signaling is not yet understood. By deactivating Gi/o as a GAP, RGS14 could function to situationally increase cAMP levels/PKA activation [10]. However, RGS14 suppression of LTP in CA2 depends, in part, on PKA inhibition [79], suggesting other factors are also important. Consistent with this idea, our most recent report demonstrates that the RGS domain of RGS14 is not essential for its inhibitory effects on LTP [19]. Therefore, RGS14 inhibits synaptic plasticity and likely PKA in a manner independent of its effect on Gi/o–cAMP signaling.

Despite not being essential for RGS14’s effects on plasticity [19], RGS control of Gi/o signaling is still important in the hippocampus and elsewhere. A number of Gi/o-coupled GPCRs are highly expressed in area CA2 [66]. Indeed, Gi/o-coupled type III metabotropic glutamate receptors (mGluR) are highly expressed in area CA2, and activation of these receptors unleashes plasticity onto CA2 [95]. While uncertain, RGS14 may modulate signaling of these metabotropic glutamate receptors in addition to other highly enriched Gi/o-coupled receptors for proper neuronal function. Finally, RGS14 is expressed in MSNs of striatum in both rodents and primates, and a loss of RGS14 in rodents increases cocaine-induced locomotion [56]. As cocaine-induced locomotion is partially dependent on various Gi/o-coupled receptors [96,97], RGS14 may limit postsynaptic signaling caused by increased monoamine stimulation in either MSNs or elsewhere to modulate locomotor responses to cocaine.

### 5.2. RGS14 Regulation of Inactive Gi1/3

RGS14 is unusual in that it also possesses a second G-protein binding site, the GPR motif, which binds inactive GDP-bound Gαi1/3 [10,11,20] (Figure 3). We propose that RGS14, following deactivating a Gai1/3 as a GAP, is captured at the plasma membrane by the Gα–GDP binding to the GPR motif [12]. Consistent with this idea, RGS14 subcellular localization at the plasma membrane in postsynaptic neurons is dependent on its binding to Gαi [12,20,98]. Indeed, RGS14 binding to either active, GTP-bound Gαi/o [12,14,16,98], or inactive Gαi1/3 [20] is sufficient to localize RGS14 to the plasma membrane. When stabilized as an RGS14–Gαi complex, RGS14 presents a local signaling nexus, capable of engaging subsequent GPCR–Gα and other signaling events. By occupying inactive Gαi1/3, RGS14 may also limit the pool of Gαi protein available for activation by GEFs [12,99]. Supporting these ideas, RGS14 is capable of simultaneously binding inactive Gαi via its GPR motif, while retaining the GAP activity of its RGS domain [12]. However, RGS14 binding to inactive Gαi1/3 does not interfere with the reformation of the heterotrimeric G-protein complex, indicating that RGS14 limits Gα signaling without prolonging Gβγ signaling [99].

With respect to RGS14 effects on LTP and synaptic plasticity, the GPR motif is essential for RGS14’s inhibitory actions in hippocampal neurons [19]. A mutation in the GPR motif of RGS14, which prevents binding to Gαi1/3, abolishes RGS14’s capacity to inhibit LTP in hippocampal slices. This suggests that plasma membrane localization is essential for RGS14’s actions, though we cannot rule out unknown signaling roles linked to inactive Gαi1/3 [100]. Related to this idea, RGS14 is a nuclear–cytoplasmic shuttling protein [20,21,98] that contains a nuclear export sequence (NES) embedded within the GPR motif [19,21]. Mutations that alter Gαi1/3 binding also disrupt RGS14 nuclear export, thereby redirecting RGS14 from the plasma membrane to the nucleus [19]. In the same report, naturally occurring, rare genetic variants in the NES sequester RGS14 in the nucleus away from the spines, thereby allowing LTP into the hippocampal neurons. Therefore, we propose that RGS14 must localize to the plasma membrane by binding to inactive Gαi1/3–GDP, via the GPR motif, to regulate glutamatergic and other signaling events and inhibit LTP.

### 5.3. Regulation of RGS14 Signaling by the 14-3-3 Scaffolding Protein

Besides binding Gαi/o proteins, RGS14 also binds other signaling proteins, including 14-3-3 [18]. The 14-3-3 family is a group of scaffolding proteins that can alter the conformation, activity, and availability of binding sites, and the localization of their interacting partners [93]. Recently, our lab and others have reported that RGS14 is a nuclear–cytoplasmic shuttling protein, and have made efforts to determine what regulates RGS14 localization in subcellular compartments [20,21,98]. In the hippocampus, 14-3-3 proteins are essential for LTP in CA1, and the inhibition of 14-3-3 impairs associative learning and memory [101]. RGS14 binds 14-3-3γ in hippocampal neurons in a phosphorylation-dependent manner at Ser218, which is located in the linker region between the RGS and R1 RBD domains. Of interest, 14-3-3 also binds RGS14 in a phosphorylation-independent manner at a distinct unknown site [18]. Phosphorylation-dependent interactions are enhanced by H-Ras activation, which results in the phosphorylation of Ser218, suggesting kinase activity downstream from H-Ras converges on RGS14 to enhance RGS14–14-3-3γ binding. However, H-Ras-dependent enhancement of RGS14–14-3-3γ binding occurs 2 h after H-Ras activation, which may reflect that RGS14 phosphorylation and the subsequent enhanced binding to 14-3-3γ first requires transcription and translation [18]. Functionally, phosphorylation-dependent 14-3-3γ binding to RGS14 inhibits RGS14 GAP activity towards GTP-bound Gαi, without affecting other RGS14 interactions. Importantly, the phosphorylation-independent 14-3-3γ interaction prevents RGS14 nuclear import. Clearly, 14-3-3γ is a key regulator of RGS14 subcellular localization and actions in hippocampal neurons important for synaptic plasticity.

### 5.4. RGS14 Regulation of Monomeric GTPase Signaling

In addition to interacting with heterotrimeric G proteins, RGS14 also binds to small, monomeric GTPases of the Ras superfamily, in particular H-Ras and Rap2A [9,16,102]. Indeed, RGS14 was first identified as a Rap2A binding partner [9]. Activated H-Ras potentiates intracellular signaling by stimulating MAPK/ERK signaling, while Rap2 inhibits MAPK/ERK signaling by shunting signaling towards the TNIK/JNK pathway [90]. Both pathways play an important role in postsynaptic signaling. H-Ras activation of ERK signaling modulates the function of AMPARs and NMDARs, trafficking and recycling of AMPARs, and structural components of the dendritic spine to modulate spine morphology [90]. Activated Rap2A opposes MAPK/ERK signaling and facilitates AMPA receptor recycling away from the membrane, causing depotentiation [103]. Therefore, H-Ras and Rap2 modulate plasticity in the postsynaptic neuron through overlapping and opposing processes, and RGS14’s interactions with each is likely important for its actions in the postsynaptic neuron.

RGS14 contains an R1/R2 domain, where activated H-Ras and Rap2 bind at an overlapping site on R1. RGS14 binding to active H-Ras inhibits MAPK/ERK signaling following receptor activation [14,16,102]. Interactions with active H-Ras localize RGS14 to the plasma membrane [14,16], and interactions with Gαi1/3 enhance RGS14–H-Ras binding [14]. RGS14 inhibits receptor-dependent ERK activation by interacting with H-Ras in PC12 cells [16,102], and RGS14 inhibits ERK signaling to block LTP in area CA2 [57]. However, the R1/R2 RBD of RGS14 is dispensable for its effects on LTP, as mutations in this domain have no effect on RGS14 suppression of LTP [19]. Although RGS14 was originally discovered as a Rap1/2 effector and is expressed in the hippocampus [9], RGS14–Rap2 interactions have been less characterized. The binding of active Rap2A to R1 does not alter RGS14 GAP activity towards active Gαi1 or RGS14 interaction with inactive Gαi1 [104]. The role of RGS14 may be to localize H-Ras/Rap2 to specific downstream effectors and/or sequester H-Ras/Rap2 away from other effectors. Future experiments will be needed to test these possibilities in the context of postsynaptic signaling and plasticity.

### 5.5. Proteins Involved in Dendritic Spine Structure

**Figure 4:**
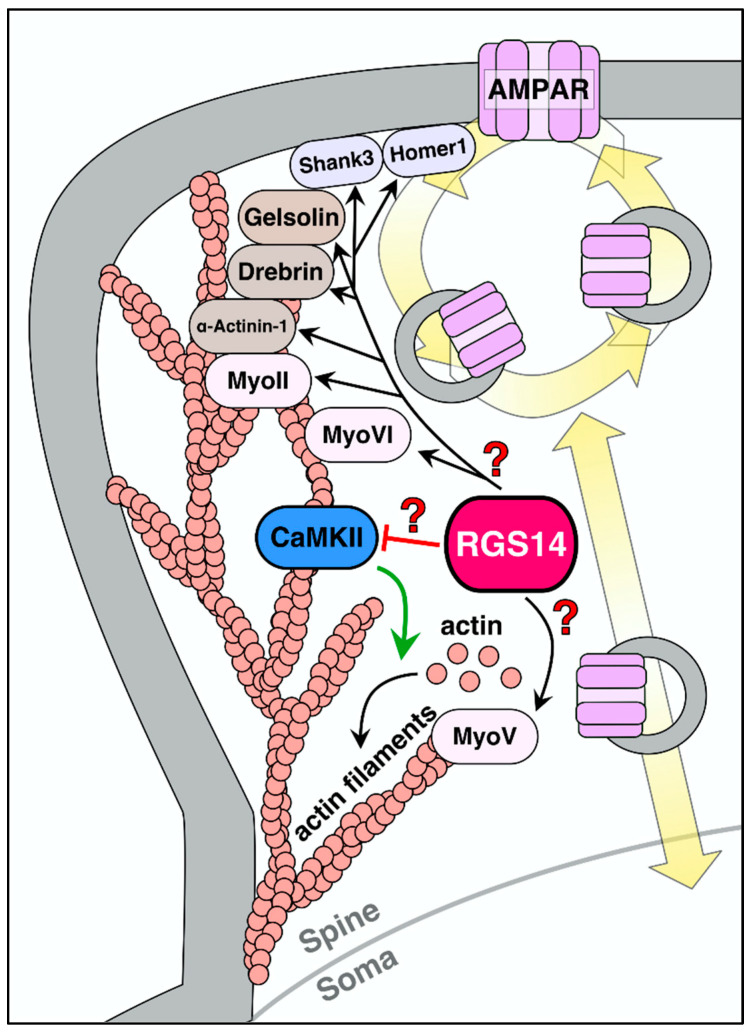
RGS14 potential regulation of dendritic spine morphogenesis. RGS14 exists in the brain as a high molecular weight complex with proteins associated with cytoskeletal architecture and dendritic spine morphogenesis, including actin-binding proteins (α-actinin-1, drebrin, gelsolin), myosins (MyoII, MyoV, MyoVI), and CaMKII. Myosins also mediate AMPAR trafficking between the plasma membrane and the soma. Additionally, human/primate RGS14 could possibly interact with postsynaptic density proteins (e.g., Homer1, Shank3) that link actin cytoskeleton to glutamate receptors and scaffold receptors to key signaling molecules.

Recent proteomic studies identified RGS14 binding interactions in the mouse brain [17]. The findings reported that RGS14, when recovered by immuno-affinity capture out of whole-brain lysates, exists in a very high molecular weight complex, suggesting that endogenous RGS14 exists as part of a larger protein complex. Recovered proteins were subjected to mass spectrometry and proteomic analysis, revealing a network of 233 proteins that were significantly enriched in complex with RGS14. Within this RGS14 interactome, the most highly enriched interactors are postsynaptic proteins involved in AMPA receptor trafficking (myosins IIb, V, and VI) [105], actin-binding proteins that maintain the dendritic cytoskeleton (e.g., drebrin, gelsolin, alpha actinin 1) [106], postsynaptic density (PSD) proteins that scaffold AMPA/NMDA receptors to key signaling molecules (Homer1, Shank3) [107], and kinases/phosphatases that transduce signals in dendritic spines (CaM, CaMKII, and protein phosphatase 1) [108]. Obviously, RGS14 does not bind all of these proteins, but likely acts on a few common proteins key to postsynaptic signaling events that support spine plasticity (Figure 4).

Among the most highly enriched proteins in the RGS14 interactome are myosins, which regulate actin dynamics and traffic cargo to and from the membrane [105]. Myh10 forms the heavy chain of the non-muscle myosin II motor protein, which helps translocate and crosslink actin filaments in dendritic spine morphogenesis [109]. Myh10 is critical for the maintenance of plasticity in area CA1, and the formation of actin filaments at activated synapses [110]. By interacting with Myh10, RGS14 could prevent actin polymerization to inhibit structural modulation of dendritic spines in area CA2 by an unknown mechanism. RGS14 is also in complex with Myo5A, which may be important for GluR1 trafficking from the Golgi apparatus to the plasma membrane in LTP induction [111], and Myo6, which regulates AMPA receptor endocytosis in hippocampal neurons [112]. However, the role of RGS14 in AMPA receptor trafficking in the postsynaptic spines of hippocampal neurons remains uncertain and is a topic of ongoing research.

RGS14 may also interact with several actin-binding proteins (ABPs) that are essential for spine remodeling during LTP [17]. Actin severing, polymerization, branching, and capping are regulated by a host of proteins, including drebrin, gelsolin, Arp2/3, and alpha actinin [106,113], which are highly enriched in the RGS14 interactome. RGS14 may be modulating the activity of one or more of these proteins to inhibit glutamate-induced spine enlargement in area CA2. Supporting a role in structural plasticity, RGS14 has been shown to bind to microtubules in the brain and regulate polymerization [114]. Furthermore, the PSD proteins Homer1 and Shank3 link receptor-mediated signaling to downstream effectors to facilitate actin remodeling and AMPA receptor insertion during LTP [115,116]. RGS14 likely acts in a similar fashion, linking membrane-bound signaling molecules and cytosolic components to prevent plasticity. Whether RGS14 inhibits transduction from the postsynaptic density, or limits actin remodeling by direct interaction with PSD proteins or ABPs, respectively, is a topic for future study.

### 5.6. RGS14 Binding to Calmodulin/Calmodulin-Dependent Kinase and Regulation of Spine Calcium

RGS14 also engages calcium signaling pathways in dendritic spines. RGS14 inhibits calcium influx from both voltage-gated calcium channels (VGCCs) [117] and ionotropic glutamate receptors [79]. More specifically, RGS14 robustly buffers calcium transients caused by glutamate uncaging in area CA2, where it is expressed, and in area CA1 when it is ectopically expressed [79]. RGS14 inhibition of calcium transients in the postsynaptic spines coincides with the suppression of LTP, and dendritic spine remodeling [79]. Furthermore, only supraphysiological concentrations of calcium, i.e., 8 mM, can reverse RGS14’s effects on plasticity in area CA2, demonstrating RGS14’s impressive calcium handling capabilities [79]. Although it is not yet known how RGS14 buffers calcium, it is possible that RGS14 suppresses channel-mediated calcium transients or increases the capacity of calcium pumps to extrude calcium from the cytosolic space. For example, both NMDAR and VGCC are regulated by Ca^2+^/CaM and CaMKII [118,119,120,121], and RGS14 could interfere with these regulatory mechanisms.

Ca^2+^/CaM and CaMKII are essential mediators of synaptic plasticity. CaM, CaMKIIα, and CaMKIIβ were identified as novel binding partners in the mouse brain from the RGS14 interactome [17]. Cell-based studies found that RGS14 was shown to directly bind CaM in a calcium-dependent manner and CaMKIIα in a calcium-independent manner [17]. Similarly, RGS14 co-localizes with CaM and CaMKIIα strongly in area CA2 and weakly in area CA1. CaM is activated by calcium transients from NMDAR and/or voltage-gated calcium channels (VGCCs) and binds to and activates CaMKII with a high concentration of intracellular calcium [122]. CaM/CaMKII phosphorylates several important targets involved in synaptic plasticity, including certain VGCCs, AMPA, and NMDA receptors, structurally related proteins such as MAP2, and signaling proteins such as cAMP response element-binding protein (CREB) [123]. CaMKIIβ binds to filamentous actin to bundle and stabilize actin filaments during plasticity events [124], and CaMKII activation is both necessary and sufficient for LTP in the hippocampus [91] and directly results in spine remodeling [87,125]. Therefore, it is possible that RGS14 regulates CaM/CaMKII signaling in some capacity to inhibit synaptic plasticity in area CA2. Indeed, LTP in area CA2 of RGS14-KO mice can be suppressed via CaMKII inhibition [79], suggesting that RGS14 inhibits CaM/CaMKII signaling by an unknown mechanism. It could be possible that RGS14 sequesters CaM and/or CaMKII away from downstream targets that would normally result in LTP, and/or scaffolds CaMKII with targets that suppress LTP. Further studies are needed to determine how RGS14 engages Ca^2+^/CaM and CaMKII to exert its actions on spine calcium.

## 6. RGS14 Regulation of Long-Term Potentiation (LTP)

RGS14 regulates synaptic plasticity measured as LTP and spine remodeling [19,57,79]. LTP has classically been separated into two general components after initial induction. One is early LTP (E-LTP), where synaptic strength is initially increased in a protein synthesis-independent manner within the first 2 h of the initial stimulation [126]. The second is late LTP (L-LTP), where nuclear transcription and protein synthesis are required to maintain the increases in synaptic strength up to 24 h after the initial stimulation [126]. LTP is first induced by coincidental activation of NMDARs by glutamate and depolarization of the postsynaptic neuron, which causes calcium transients to enter into the cell [127]. When sufficiently high amounts of calcium enter the postsynaptic neuron, calcium activates CaM, which in turn binds to CaMKII, causing CaMKII autophosphorylation and activation [122].

The active translocation of CaMKII to synapses is necessary and sufficient for E-LTP by phosphorylating a host of downstream targets [91,125,128]. Of note, RGS14 binds Ca^2+^/CaM and CaMKII and is phosphorylated by CaMKII [17]. Phosphorylation of NMDARs by CaMKII enhances single-channel conductance and is necessary for LTP [129], and the phosphorylation of AMPARs and transmembrane AMPAR-regulatory proteins (TARPs) by CaMKII causes lateral diffusion, insertion, and stabilization of AMPARs into PSD slots to increase postsynaptic potentiation [130,131]. Endosomes containing AMPARs are exocytosed by the actions of many different proteins, including myosins [110,132], thereby increasing the rate of AMPAR exocytosis vs. endocytosis and increasing postsynaptic potentiation. RGS14 engages myosins in the brain as part of a high molecular weight complex [17]. CaMKII also phosphorylates and activates a set of GEFs and GAPs for monomeric regulatory GTPases, which cause actin polymerization and spine remodeling to produce what is known as structural plasticity [133]. RGS14 directly binds activated H-Ras and Rap2A, which are likely to modulate these events [9,14,15,16,18,102,104]. Although both functional and structural plasticity during E-LTP are critical for memory formation, AMPAR-mediated EPSCs are necessary for E-LTP, while spine remodeling is not and is mechanistically distinct from the E-LTP mechanisms [134].

E-LTP transitions to L-LTP, as signals from the dendritic spines are transduced to the nucleus, causing the activation of transcription factors. In turn, newly synthesized, plasticity-related RNAs and proteins are translocated to the activated synapse to maintain potentiation [135]. RGS14 shuttles between the cytoplasm and nucleus, where it could engage with key signaling partners to regulate the mechanisms of L-LTP [18,19,20,21,98]. Of note, stimulation of PKA and MAPK/ERK result in CREB activation, which causes the transcription of CRE-driven genes that maintain plasticity [136]. Importantly, inactivating mutations in PKA and CREB impair L-LTP, but not E-LTP [137,138]. Given that RGS14 inhibits both PKA and MAPK/ERK signaling to suppress E-LTP [57,79], it is likely that RGS14 would engage with G-protein-dependent and/or -independent processes to suppress L-LTP. Additionally, local translation of mRNA at dendritic sites of activation is emerging as a key player in maintaining the functional and structural modifications of synapses [113,139]. A variety of key plasticity proteins, such as CaMKII, are locally translated [113], and disruption of the local translation of CaMKII impairs L-LTP and spatial memory [140]. Fascinatingly, RGS14 mRNA is readily available at both CA1 and CA2 dendrites and cell bodies [141], suggesting RGS14 expression may be activity-dependent, similar to RGS2 [142]. Therefore, RGS14 may exert further control of L-LTP by being expressed in an activity-dependent manner and regulating signaling from locally translated proteins.

### 6.1. RGS14 Regulation of E-LTP and Potential Mechanisms

**Figure 5:**
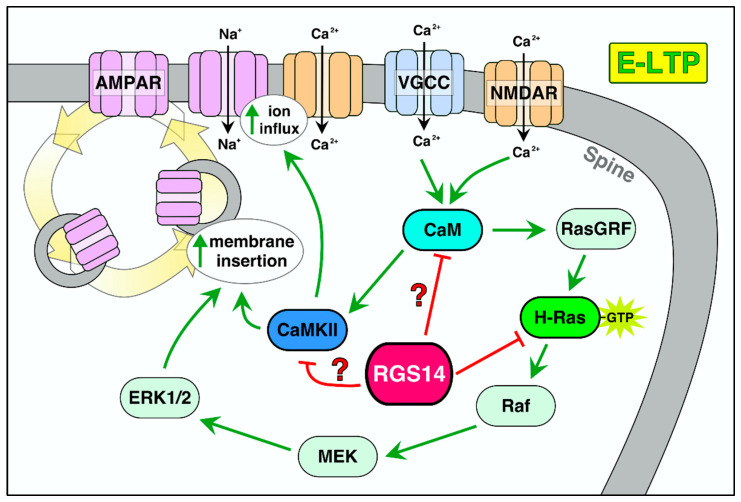
RGS14 regulation of early LTP. RGS14 limits Ca^2+^ signaling during LTP induction, potentially through direct interaction with Ca^2+^/CaM and/or CaMKII. RGS14 also directly binds activated H-Ras–GTP and Ca^2+^/CaM likely to dampen ERK/MAPK signaling. RGS14 inhibition of Ca^2+^ and MAPK signaling may curtail CaMKII- and ERK1/2-mediatied AMPAR membrane insertion and AMPAR and NMDAR channel potentiation to inhibit early long-term potentiation (E-LTP).

By engaging key signaling proteins and pathways essential for spine signaling and plasticity, RGS14 is well positioned to block E-LTP at the spines where it is present (Figure 5). We have demonstrated that RGS14 inhibits potentiation of the postsynaptic response in area CA2 dendrites after Schaffer collateral stimulation [19,57,79]. Our recent studies show that RGS14’s inhibitory actions on post synaptic plasticity extend to inhibiting structural changes within single dendritic spines [79]. Yet, RGS14 is also capable of inhibiting plasticity in other neurons. The ectopic overexpression of RGS14 in hippocampal area CA1 pyramidal cells suppresses postsynaptic potentiation and structural plasticity there as well [19,79], suggesting RGS14 acts on conserved mechanisms shared by neurons in areas CA1 and CA2 to control LTP. Consistent with this idea, RGS14 directly engages G proteins, H-Ras/Raf/ERK and Ca^2+^/CaM/CaMKII signaling pathways, and the actomyosin system to regulate spine plasticity.

RGS14 blocks LTP in both CA1 and CA2 after high-frequency stimulation during the early phase of LTP (between 0 and 30 min following stimulation). RGS14 inhibits both synaptic potentiation and structural plasticity during this time period, indicating that RGS14 prevents the expression of E-LTP. Genetic mutations in the GPR motif that prevent RGS14–Gαi binding perturb RGS14 plasma membrane localization, thereby blocking RGS14 suppression of LTP [19]. Therefore, RGS14 must localize at the plasma membrane to carry out its actions on LTP. How could RGS14 be preventing E-LTP? Our lab has shown that the unmasked LTP in CA2 due to the loss of RGS14 (RGS14-KO) can be suppressed by pharmacologically inhibiting CaMKII, NMDAR, PKA, and MEK/ERK [19,57,79]. Thus, RGS14 may act directly or indirectly on these targets to suppress LTP.

RGS14 inhibits calcium transients from NMDAR and L-type VGCCs [79,117]. Since calcium influx in spines is essential for the activation of RGS14 binding partners, post synaptic signaling and LTP, the RGS14 blockade of the spine calcium could block the activation of calcium-dependent CaM, CaMKII, and Ras/MEK/ERK, all of which are critical for the expression of E-LTP [125,143]. Additionally, RGS14 buffering of calcium correlates with its blockade of spine enlargement, and increasing extracellular calcium allows for spine enlargement in RGS14-positive spines, further implying RGS14’s control of calcium is key to its inhibition of plasticity. However, it is unknown how RGS14 inhibits calcium influx from NMDARs or VGCCs. RGS14 does not possess a calcium-binding domain, and there is no evidence that it binds to NMDARs or VGCCs. In fact, NMDAR and AMPAR currents in area CA2 are similar between RGS14 WT and KO mice when stimulated with a stimulus that does not evoke LTP [57]. Though speculation, RGS14 may modify calcium currents indirectly by engaging proteins that regulate channel function.

In addition to inhibiting calcium influx, RGS14 likely interferes with calcium-dependent signaling pathways. RGS14 could block spine signaling by serving as a local sink/buffer for key proteins/pathways. For example, RGS14 could bind to CaM after glutamate-induced calcium influx to prevent CaM from interacting with plasticity-inducing proteins [17]. Indeed, RGS14 and CaM share an overlap in their profile of binding partners and involvement in a variety of signaling pathways [144]. Both proteins interact with CaMKII, myosin light chain and tubulin, and both modulate VGCC activity, cAMP concentration, and Ras activation. CaM-regulated adenylyl cyclases AC1 and AC8 are expressed in the hippocampus and facilitate LTP in this region [145,146,147,148]. Upon CaM activation and subsequent binding to AC1/8, intracellular cAMP levels increase, which activate PKA [145]. RGS14 could suppress plasticity by sequestering CaM away from AC1/8 and blocking downstream PKA activation. In a similar manner, RGS14 may inhibit CaMKII signaling by inhibiting upstream calcium signals, and binding CaMKII directly in a calcium-independent manner [17]. Alternatively, RGS14 could prevent CaM from binding to CaMKII by modulating upstream calcium or sequestering the Ca^2+^/CaM/CaMKII complex from target proteins that are essential for E-LTP, such as AMPARs, NMDARs, or plasticity-related scaffold proteins [125].

Another possible mechanism by which RGS14 inhibits E-LTP would be its interactions with active H-Ras. Calcium influx and CaMKII can activate H-Ras and cause downstream ERK activation, which phosphorylates AMPAR to promote the insertion of AMPARs into the PSD [149,150]. Our lab and others have shown that RGS14 binds to active H-Ras to inhibit ERK phosphorylation and regulate neurite outgrowth in PC12 cells [14,16], and RGS14 effects on ERK signaling are at least partially responsible for its suppression of LTP in area CA2 [57]. However, we recently reported that mutations in the R1 RBD of RGS14 that mediate H-Ras binding do not affect RGS14’s ability to suppress LTP [19]. Therefore, RGS14 likely regulates E-LTP by modulating calcium, CaM, and/or CaMKII signaling pathways upstream of H-Ras activation. However, RGS14 interactions with H-Ras and/or Rap2A likely have functional consequences on postsynaptic signaling, L-LTP and heterosynaptic plasticity [143,151].

### 6.2. Potential Roles for RGS14 in L-LTP and in the Nucleus

**Figure 6:**
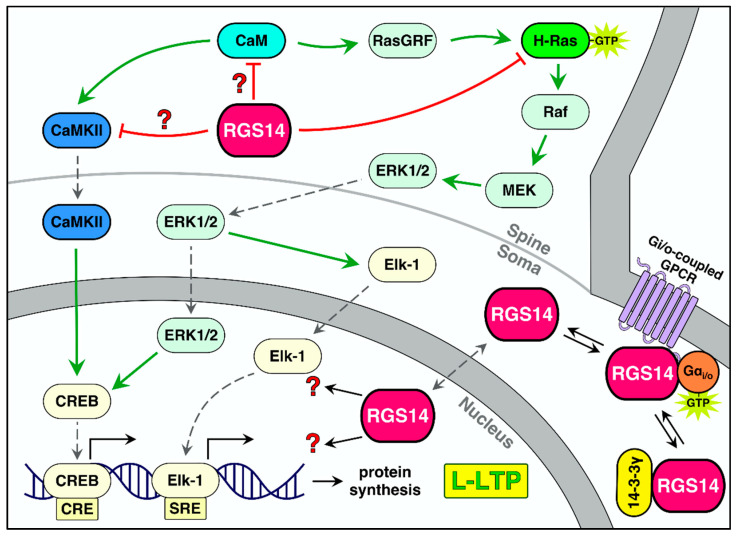
RGS14 regulation of late LTP outside and within the nucleus. RGS14 suppression of Ca^2+^ and MAPK signaling could potentially curb CaMKII- and/or ERK1/2-stimulated transcription from CRE and SRE sites, which drive expression of proteins that stabilize synaptic strengthening to effect late long-term potentiation (L-LTP). Cytoplasmic RGS14 shuttles in and out of the nucleus. In the nucleus, RGS14 localizes to both chromatin-rich and chromatin-poor subregions. The function of RGS14 in either subregion is not known, but nuclear RGS14 could potentially modulate L-LTP in some manner. The subcellular localization of RGS14 is in a dynamic balance influenced by available binding partners. Gαi/o-bound RGS14 is found at the plasma membrane, while 14-3-3γ binding blocks nuclear import and sequesters RGS14 in the cytoplasm.

The induction of LTP is directly linked to memory formation [61], and memories must be consolidated to establish long-term memory. L-LTP maintains increases in the synaptic strength of activated synapses over the course of days and longer [135]. Therefore, intact L-LTP is required for hippocampal-dependent long-term memory. Although RGS14 clearly suppresses short-term plasticity (i.e., E-LTP), there may be a role for RGS14 in L-LTP as well (Figure 6). The most compelling evidence for a role of RGS14 in L-LTP is the apparent enhancement of spatial learning in RGS14-KO mice compared to WT mice [57]. In the Morris water maze (MWM) assay of spatial memory, days after the initial exposure, RGS14-KO mice locate the hidden platform quicker than WT mice. This effect is most apparent at later days in the task. In a novel object recognition task, RGS14-KO mice, compared to WT mice, have increased preference for a novel object over familiar ones 4 h after initial exposure to the familiar objects. These two pieces of data suggests that a loss of RGS14 may enhance L-LTP to better preserve object and spatial memory. How could RGS14 regulate L-LTP?

A recent report from our lab sheds light on this question [19]. As discussed previously, RGS14’s localization is essential to its plasticity-resistant properties, and RGS14 is localized not only to the dendrite and spines, but is also enriched in the soma of CA2 neurons [26,27,57]. Of note, RGS14 is a dynamic protein that shuttles between the membrane, cytosol, and nucleus of host cells [20,21,98]. We show that a human genetic variant of RGS14, L504R, which is located within the NES, sequesters RGS14 in the nucleus. When expressed in the hippocampus, the L504R variant behaves similarly to RGS14-KO by allowing for robust E-LTP. We used CRISPR/Cas9 to introduce this L504R mutation in the NES of RGS14. To our surprise, RGS14-L504R mice performed similarly to WT mice in the MWM task, despite the ability to express E-LTP. This difference in RGS14-KO and RGS14-L504R mice suggests that RGS14 could localize to the nucleus to interrupt L-LTP and prevent memory enhancement that exists in RGS14-KO mice. That is, loss of dendritic RGS14 is sufficient to restore E-LTP, but parallel loss of nuclear RGS14 may also be necessary for effects on L-LTP and long-term memory enhancement.

**Figure 7:**
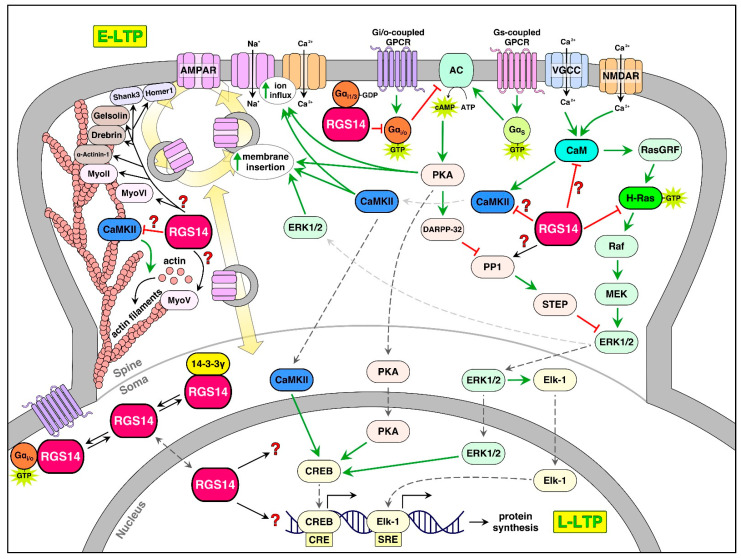
RGS14 modulation of postsynaptic signaling and plasticity. RGS14 binds many protein partners, directly or indirectly, to regulate signaling events and plasticity in the post synaptic dendritic spine (top) and in the soma where it shuttles in/out of the nucleus (bottom). Key: green line with arrow = activation (increase if inside bubble), red line with T arrow = inhibition, black line with arrow = interaction or relationship (unspecified directionality), dashed gray line = movement, question mark = unknown or uncertain interaction. This key is also used in Figure 3, Figure 4, Figure 5 and Figure 6.

RGS14 has been observed in the nuclei of striatal neurons of monkey [26], though the roles of RGS14 in the nucleus remain unclear at present. RGS14 is naturally expressed in B35 neuroblastoma cells, where it localizes to chromatin-poor and chromatin-rich regions, nuclear pore complexes, along the nuclear envelope, and in juxtanuclear membranes outside the nucleus, and a subset of RGS14 localizes with active RNA polymerase II [98]. Whether RGS14 alters transcription in stimulated neurons is unknown. A pilot study of WT RGS14 vs. nuclear sequestered RGS14-L504R expressed in resting hippocampal neurons found no differences in transcription at resting state [19]. In contrast, we show that immediate early gene (IEG) c-Fos is increased in RGS14-KO mice compared to RGS14-WT mice 90 min after a high dose of cocaine [56], suggesting that RGS14 suppresses nuclear IEG expression in stimulated neurons. This may be due to RGS14 regulation of CaM/CaMKII-, Gi/o-, ERK-, and PKA-dependent signaling outside and within the nucleus, which is likely to suppress the transcription and translation necessary for L-LTP. Another possibility is that RGS14 may regulate trafficking of interacting partners to and from the nucleus, and/or regulate signaling within the hippocampus. By shuttling between dendritic spines and the nucleus, RGS14 could regulate the transport of plasticity-related proteins back and forth to alter LTP (Figure 6). Future experiments will elaborate on functional roles of nuclear RGS14 after LTP induction. A summary model of RGS14 regulation on postsynaptic signaling and synaptic plasticity can be found in Figure 7 below.

### 6.3. Modulation of RGS14 Inhibition on CA2 Plasticity

Of interest is the curious fact that induction of LTP markedly differs within distinct dendrites of the same CA2 neuron that express RGS14. In proximal dendrites, which receive CA3–CA2 synapses from the stratum radiatum (SR), LTP is absent, but can be elicited with pharmacological modulation. By contrast, distal dendrites of the stratum lacunosum-moleculare (SLM), which receive strong, excitatory input from entorhinal cortex layers II and III, express robust LTP [66]. Here, we discuss how RGS14 could be modulated to differentially facilitate LTP at the dendritic spines where it is expressed. 

Pharmacological stimulation and inhibition of several different GPCRs in CA2 can restore LTP in neurons containing RGS14. The activation of vasopressin 1b receptor (Avp1br), oxytocin receptor (Oxtr), and substance P receptor are sufficient to reverse RGS14 inhibition of LTP [152,153,154]. Likewise, antagonism of adenosine 1a receptors and type III metabotropic glutamate receptors produces a similar effect on CA2 LTP [95,155,156]. How might RGS14 actions be inhibited to promote plasticity?

Regulation of RGS14 subcellular localization could be a key factor. Mutations altering RGS14 localization out of the spines prevent the inhibition of LTP, specifically those that prevent membrane localization and promote nuclear sequestration [19]. It is possible that posttranslational modification downstream of receptor stimulation would alter RGS14 localization. Indeed, RGS14 is phosphorylated by PKA [157], protein kinase C (PKC) [158], CaMKII [17], ERK [158], and by an unknown kinase downstream of H-Ras [18], although the functional consequences of these modifications on RGS14 actions in spines remain undefined. Furthermore, LTP restoration by inhibition of the Gi/o-coupled adenosine 1a receptor and type III metabotropic glutamate receptors in area CA2 suggests tonic activation of these receptors. It is possible that constitutive Gi/o-signaling in CA2 dendrites retains RGS14 at the plasma membrane of spines, where RGS14 functions to suppress LTP. Consistent with this idea, RGS14 is recruited to the plasma membrane by Gi/o-linked GPCR activation or inactive Gαi1/3 [14,20]. Therefore, constitutively active Gi/o-linked GPCRs could retain RGS14 at the membrane to suppress LTP, and inhibition of these receptors could dissociate RGS14 from the plasma membrane.

Additionally, activation of Gαq-coupled oxytocin or vasopressin 1b receptors on CA2 pyramidal cells causes downstream PLC-dependent calcium influx [152,153], which increases synaptic potentiation in CA2. One possibility is that Gαq activation in CA2 overcomes RGS14 suppression of LTP, by causing sufficient calcium influx independent of NMDAR activation and activating downstream PKC [159], which promotes AMPAR-mediated potentiation [160]. Supporting this, a recent report demonstrated that NMDA receptors in vasopressin 1b-expressing cells (primarily area CA2) are not required for vasopressin 1b receptor-dependent behaviors, such as social aggression [161]. Therefore, there may be Gαq-dependent mechanisms within CA2 that bypass and overcome RGS14 LTP inhibition.

Lastly, while proximal dendrites of the SR are resistant to LTP, distal dendrites of the SLM readily undergo LTP after activation of layer II/III EC afferents to CA2 [67,95,154]. RGS14 is expressed throughout the pyramidal cells of area CA2, including the dendrites of the SR and SLM [26,27,57]. If RGS14 is expressed in both sets of dendrites, why does RGS14 only inhibit plasticity in the SR? At this time, there are no studies that have investigated molecular differences between LTP-resistant proximal dendrites and LTP-supporting distal dendrites. Future studies will need to be performed to understand the mechanisms by which RGS14 acts on at each set of CA2 spines.

## 7. Roles for RGS14 in Hippocampal Behavior

As discussed earlier, RGS14-KO mice exhibit enhanced spatial memory in the MWM task and enhanced novel object recognition [57], both of which are hippocampal-dependent behaviors [162,163]. To date, these are the only hippocampal-dependent behavioral tasks reported for RGS14-KO mice. A recent report demonstrated that female, but not male, RGS14-KO mice have an enhanced fear response in a cued fear conditioning paradigm [164]. This effect was only seen two weeks after cued fear conditioning, suggesting RGS14 in the hippocampus may play a role in consolidating the cued fear response. Contrary to this point, most studies assert that the hippocampus is required for contextual fear conditioning, while the amygdala is required for cued fear [165]. Related to this, our lab has shown that RGS14 is expressed in the central amygdala of rodents and primates [19,26]. Therefore, a more plausible explanation for the enhanced cued fear in female RGS14-KO mice may be due to enhanced plasticity in the central amygdala rather than enhanced CA2 plasticity.

Why would unregulated CA2 LTP enhance hippocampal-dependent memory? In the MWM task, NMDAR-dependent CA3–CA1 LTP is required for learning [89], and optogenetic inactivation of CA2 has no effect on spatial learning [68], suggesting CA2 activity is not required for the acquisition of spatial memory. One possibility is that CA2 LTP enhances potentiation in area CA1. During spatial learning, CA3 pyramidal cells are activated and fire onto area CA1 [61]. Similarly, area CA3 projects onto CA2 and likely transmits to CA2 in a similar manner. Normally, RGS14 would prevent recruitment of CA2 into this circuit, but the loss of RGS14 allows the expression of CA2 LTP and the recruitment of CA2 into the circuit. CA2 pyramidal cells project strongly onto the stratum radiatum and stratum oriens of area CA1 [66,67]. Enhanced excitatory input from CA3 and CA2 onto CA1 could strengthen CA1 synapses moreso than with CA3 input alone. Although our lab has shown that CA1 LTP in RGS14-KO mice is normal during high-frequency stimulation of Schaffer collaterals, how CA2 LTP in RGS14-KO mice affects CA1 plasticity in vivo has yet to be determined.

A truncated form of human RGS14 (RGS_414_) has been investigated for its effects on rodent behavior linked to hippocampal memory [166,167]. Lentiviral expression of RGS_414_, in layer six of the V2 visual cortex of rats, enhances object recognition memory and permits long-term memory of a novel object months after initial exposure [167]. In a separate report, the introduction of RGS_414_ into area CA1 of rats enhanced neurite formation and branching in area CA1 pyramidal cells, which was dependent on increases in BDNF production [166]. RGS_414_ expression in CA1 also enhanced Y-maze and MWM performance in adult, aged, and AD-model rats.

These findings with an overexpressed truncated form of RGS14 conflict with previous reports showing that a loss of endogenous RGS14 enhances hippocampal-dependent spatial memory in mice. In related findings, expression of full-length rat RGS14 and an RGS-null mutant in area CA1 of mice restricts LTP [19,79], which would likely impair memory. Several possibilities could explain these discrepancies. This truncated form of human RGS14 (RGS_414_) is not observed to be naturally expressed in rodents or at detectable levels in the hippocampus of primates [26,27]. Furthermore, RGS_414_ lacks an RGS domain and part of the adjacent linker region, suggesting that a loss of binding to plasticity-relevant G proteins could effectively reverse this process. However, this is unlikely since an RGS-null point mutant of RGS14 suppresses LTP [19]. RGS_414_ encodes the human sequence containing a PDZ-binding ligand (DSAL), which is absent in the RGS14 sequences of rodents [44]. Therefore, it is possible that overexpressing this truncated form of RGS14 disrupts key postsynaptic density proteins with a PDZ domain (e.g., PSD95, Homer1, etc.). Although these findings with truncated RGS_414_ reflect the actions of a protein not normally found in hippocampal neurons, they are useful for revealing insights into the potential mechanisms of RGS14 actions by perturbing molecular systems in those neurons.

## 8. Roles for RGS14 in Non-Hippocampal Brain Regions

Recent reports indicate that RGS14 is also expressed in specific brain regions outside of the hippocampus [19,26,56], where its involvement in neuronal function and behavior is less well understood.

### 8.1. Amygdala

RGS14 is expressed in the amygdala of rodents and primates [19,26,56]. The amygdala is involved in emotional modulation of learning and memory, and is a key mediator of the emotional states of fear and anxiety [168]. Evidence supports a suppressive role of RGS14 in amygdala activity and amygdala-dependent behaviors. GWAS revealed a non-synonymous coding SNP in the RGS14 gene, which was associated with freezing to both cued and contextual fear conditioning [169]. In another fear conditioning study, genetic deletion of RGS14 enhanced freezing during a cued fear memory test in female mice [164]. A recent study examined the role of RGS14 in locomotor responses to novelty and cocaine [56]. Genetic deletion of RGS14 results in novelty-induced hypolocomotion and high-dose cocaine-induced hyperlocomotion. In RGS14-KO mice, both novelty and high-dose cocaine enhanced thigmotaxis (propensity for movement around the periphery of an open field vs. the center), which is an index of anxiety. This suggests that loss of RGS14 augments neophobia (novelty-induced anxiety) and the anxiogenic effect of high-dose cocaine. Additionally, a more robust induction of c-Fos and phosphorylated ERK (p-ERK) was observed in the central nucleus of the amygdala and hippocampus in RGS14-KO mice treated with cocaine in a novel environment, when compared to control mice under the same conditions. Increases in c-Fos and p-ERK induction in the central amygdala of RGS14-KO mice compared to WT mice suggests RGS14 inhibits upstream signaling and potentially plasticity in this region as well, although further studies must be performed to confirm this.

### 8.2. Ventral Striatum

RGS14 is expressed in the ventral striatum, a brain structure that sits at the limbic–motor interface where these neurons command motivation, reward processing, and goal-directed behavior [170]. It is also critically involved in psychostimulant augmentation of locomotor activity [171]. RGS14-KO mice exhibit enhanced cocaine-induced locomotor activation, suggesting that RGS14 tempers ventral striatal activation. However, loss of RGS14 has no effect on c-Fos or p-ERK induction within the nucleus accumbens after cocaine treatment in a novel environment [56]. This observation could be due to a washout of the cocaine effect in the ventral striatum by the novelty effect in the amygdala. However, little is known about the roles of RGS14 in ventral striatal function, and this is a topic for further study.

### 8.3. Basal Ganglia

RGS14 is also expressed in striatal projection neurons comprising both the direct and indirect pathways of the basal ganglia, where it demonstrates postsynaptic and nuclear localization in the striatum and presynaptic localization in the globus pallidus and substantia nigra pars reticulata [26]. The basal ganglia are implicated in habitual learning, voluntary movement, and fine motor control [172,173]. Mice lacking RGS14 exhibit normal baseline locomotor activity [56,57], but this is the extent of the current knowledge of RGS14 in basal ganglia function. Considering that RGS14 shows robust expression with diverse subcellular localization in striatopallidal and striatonigral neurons, its role in the basal ganglia presents a rich opportunity for new discovery.

### 8.4. Link to Synaptic Plasticity outside of Hippocampus

RGS14 inhibits hippocampal-based learning and memory by suppressing LTP in area CA2 by engaging and integrating G protein, H-Ras/ERK and calcium signaling pathways [57,79]. Various forms of learning and memory in the amygdala and striatum also are mediated by NMDAR-dependent LTP, which depends on ERK and calcium, including fear conditioning and reward-based instrumental learning [174]. All regions expressing postsynaptic RGS14 also receive extensive dopaminergic innervation [175,176,177], where dopamine bidirectionally modulates synaptic strength through two opposing families of receptors [178]. A growing list of RGS proteins are known to interact with dopamine signaling [179,180,181,182,183]. The observed hypersensitivity of RGS14-KO mice to cocaine [56], a potent enhancer of synaptic dopamine, suggests that RGS14 could be added to that list. RGS14 may restrict the underlying plasticity of learning and memory in multiple brain regions through shared signaling mechanisms. However, some disparities likely exist between regions due to differences in connectivity and protein expression patterns. Further investigation is required. Additionally, how RGS14 may affect presynaptic signaling in striatopallidal and striatonigral terminals is unclear. RGS14 could potentially negatively modulate cannabinoid-1 receptor-Gi/o signaling to suppress endocannabinoid-mediated LTD [181]. RGS14 may also indirectly block Gβγ-mediated inhibition of L-type calcium channels to enhance neurotransmitter release, akin to the actions of other presynaptic RGS proteins [184,185,186]. Future studies will examine these and other possible functions.

### 8.5. Implications of RGS14 outside of the Hippocampus

RGS14 is found most prominently in the brain regions comprising the limbic system and the basal ganglia. When considered hierarchically, the expression pattern of RGS14 suggests an overarching role in modulating the degree to which the emotional significance of a stimulus permits the shaping of behavior. For example, a car crash victim may experience panic attacks when driving by the crash site, and thus may deliberately choose driving routes that avoid that location. This has clear implications for the treatment of psychiatric disorders characterized by high emotional reactivity, including post-traumatic stress disorder and anxiety disorders. Multiple intervention points exist in the course of substance use disorder/addiction, in which RGS14 manipulation could be beneficial. The period of withdrawal following cocaine exposure is characterized by LTP induction of specific afferents onto ventral striatal projection neurons, and is thought to contribute to the escalation of drug use [187,188,189]. The dorsal striatum is progressively recruited as drug use becomes habitual, and anxiety-provoking situations (amygdala-mediated) and drug-associated contexts (hippocampus-mediated) are prime triggers for relapse [181,190,191,192]. Additionally, targeted manipulation of RGS14 in the basal ganglia has treatment potential for movement disorders, such as Parkinson’s disease and essential tremor [181].

## 9. Impact of Genetic Variation on RGS14 Function and Physiology in Brain

Large-scale genome and exome sequencing data have generated new information regarding the link between rare human variants, and their impact on disease predisposition and individual traits [193]. Remarkably, full sequencing of human genomes show an average of ~4 million genetic variants in any given individual [194]. One recent report describes how human genetic variants located in the nuclear export sequence of RGS14 impact protein function in hippocampal neurons [19]. RGS14 variant L505R (L504R in rodents) is found in 0.006% of the East Asian population, while R507Q (R506Q in rodents) is found in 1.25% of the Ashkenazi Jew population [193]. Notably, variant L504R completely blocks RGS14–GPR motif binding to Gαi1, while R506Q causes a reduced interaction with Gαi1 [19]. Wild-type RGS14 is localized to the soma, dendrites and spines of rat hippocampal neurons. By contrast, RGS14-L505R is concentrated in the nucleus, and R507Q displays a mixed phenotype. In CA1 hippocampal neurons, where WT-RGS14 completely blocked LTP, R506Q allowed a short-lasting potentiation, and L504R completely blocked RGS14’s capacity to inhibit LTP [19]. These findings show that genetic variants can profoundly impact protein function, and further studies are in progress to determine the impact of these variants on RGS14’s functions in rodent behaviors.

As mentioned above, human RGS14 is also expressed outside the brain, in various tissues including kidney, and differs from the rodent protein by expressing a 22 amino acid tail containing a PDZ-binding ligand (DSAL). Many GWAS report a linkage of the RGS14 gene and chronic kidney disease [33,34,35,36,37,38,39,41,42]. One recent study shows that human RGS14 directly binds NHERF1 [44], a PDZ scaffolding protein that regulates many surface receptors and transporters in the brain, kidney, and elsewhere. This study shows that RGS14 binds directly to the NHERF1–NPT2A complex to disrupt hormone-sensitive phosphate uptake in the kidney. The phosphate transporter NPT2A is found only in the kidney, but NHERF1 binds many receptors and transporters in the brain [195]. Therefore, human RGS14 likely regulates NHERF1 and other PDZ proteins in the CNS. Of note, a rare human variant was identified (D563N), located within the DSAL PDZ ligand, which disrupts RGS14 binding to NHERF1 to block its actions in the kidney [44]. RGS14–NHERF1 interactions and disruptive variants likely occur in the brain as well, and this remains a topic for further study. Undoubtedly, other genetic variants of RGS14 exist that alter protein function to impact disease predisposition and individual traits. Together, these observations show that naturally occurring genetic variants for RGS14 and other proteins can have a profound impact in neurophysiology.

## 10. Unanswered Questions and Future Directions for the Study of RGS14

Since RGS14 was first identified over 20 years ago as a Rap2-binding protein [9], much has been learned about the protein’s unusual and important role as a master regulator of synaptic signaling and plasticity. However, much remains to be learned, and here we address unanswered questions about RGS14’s roles in neurobiology.

### 10.1. A Nuclear Function for RGS14?

Quite unusual for a large scaffolding protein that regulates cell surface events, RGS14 is a nucleocytoplasmic shuttling protein. Numerous studies show that RGS14 dynamically shuttles from the cytosol to the nucleus and back [20,21,98], and regulation of its subcellular localization likely plays a major role in RGS14’s control of synaptic plasticity [19]. In related findings, mice that have nuclear sequestered RGS14 do not exhibit enhanced spatial memory, such as RGS14-KO mice [19], suggesting that nuclear RGS14 may regulate components of L-LTP. The roles for RGS14 in the nucleus are unclear, but possibilities include regulation of gene transcription and/or splicing, which could occur directly or indirectly. Alternatively, RGS14 may use the nucleus as a “timeout” to regulate its availability, but without having to be degraded and synthesized as a new protein. Future studies will define novel roles for RGS14 in the nucleus.

### 10.2. Modulation of RGS14 Activity

Additionally, it is unknown how RGS14 is regulated to engage or disengage in plasticity-related events. RGS14 is post-translationally modified via cAMP–PKA, Ca^2+^/CaMKII, PKC and H-Ras/ERK signaling [17,18,157,158]. Likewise, 14-3-3γ binds to RGS14 to control its subcellular localization, and this interaction can occur in a phosphorylation-dependent manner, enhanced by H-Ras activity [18]. Furthermore, PKA-dependent phosphorylation of RGS14 at a site near the GPR motif enhances interactions with inactive GDP–Gαi1 [157]. These findings demonstrate that RGS14’s function is effectively modulated by phosphorylation. However, several questions remain. How does CaMKII-directed phosphorylation of RGS14 affect its function, specifically relating to plasticity? Additionally, does posttranslational modification of RGS14 alter its capacity to inhibit synaptic plasticity? It seems likely that postsynaptic receptor signaling could release RGS14’s blockade of plasticity in area CA2. As discussed earlier, a number of pharmacological manipulations restore CA2 LTP when RGS14 is present. It is possible that downstream signaling from receptor activation (or antagonism in the case of the constitutively active adenosine 1a receptor and type III mGluRs) promotes posttranslational modification of RGS14, thereby causing RGS14 to be inactivated. Based on our recent report of the link between RGS14 subcellular localization and plasticity [19], phosphorylation at one or multiple sites of RGS14 could regulate its subcellular localization and disinhibit plasticity in RGS14-positive neurons.

### 10.3. RGS14 as a PDZ Scaffold Protein in the Brain

As discussed earlier, human/primate RGS14 contains a C-terminal PDZ ligand that is lacking in the rodent protein [44]. NHERF1 binds to human, but not rodent RGS14, suggesting a human-specific PDZ interaction between the two proteins. Interestingly, proteins containing PDZ domains (e.g., PSD95, Shank3, Homer1) are highly enriched in the PSD of dendritic spines [196]. PDZ proteins in the PSD regulate AMPA receptor insertion and stabilization, and scaffold membrane proteins, signaling molecules, and cytoskeleton components to promote plasticity [88,107,134]. It is therefore likely that human RGS14 interacts with additional partners in dendritic spines via PDZ-specific binding, and human RGS14 may attain new functions related to plasticity because of this binding. The report that a truncated form of RGS14 containing the DSAL motif enhances plastic processes and memory raises the question of whether RGS14 PDZ interactions could change RGS14 from plasticity inhibiting to plasticity enhancing. Future studies will focus on how full-length human RGS14, containing the PDZ ligand, modulates plasticity in the hippocampus and, if so, what protein(s) does RGS14 interact with in a PDZ-specific manner?

### 10.4. Beyond Learning and Memory: RGS14 outside the Hippocampus

Finally, what functions does RGS14 serve in brain outside of the hippocampus? Recent studies show that RGS14 is also expressed in the dorsal striatum, nucleus accumbens, and the central amygdala [19,26]. As discussed above, an increased response to cued fear conditioning in RGS14-KO mice may reflect the plasticity-related role of RGS14 in the central amygdala [164]. New studies show that RGS14 regulates novelty- and cocaine-induced locomotion, possibly by regulating postsynaptic processes in the nucleus accumbens [56]. Other RGS proteins, such as RGS9 and RGS12, mediate acute and rewarding effects to psychostimulants [180,182]. RGS14 in the dorsal striatum, nucleus accumbens, or hippocampus may play an active role in regulating similar effects. Future studies will investigate the role of RGS14 in mediating rewarding effects of psychoactive drugs, such as cocaine. While much has been learned over the past two decades about the role of RGS14 in the brain as a central regulator of postsynaptic signaling and plasticity, much more awaits discovery.
